# Histopathology, microbiology and the inflammatory process associated with *Sarcoptes scabiei* infection in the Iberian ibex, *Capra pyrenaica*

**DOI:** 10.1186/s13071-017-2542-5

**Published:** 2017-12-04

**Authors:** José Espinosa, Arián Ráez-Bravo, Jorge R. López-Olvera, Jesús M. Pérez, Santiago Lavín, Asta Tvarijonaviciute, Francisco J. Cano-Manuel, Paulino Fandos, Ramón C. Soriguer, José Enrique Granados, Diego Romero, Roser Velarde

**Affiliations:** 1 Departamento de Biología Animal, Vegetal y Ecología, Universidad de Jaén, Campus Las Lagunillas s/n, 23071 Jaén, Spain; 2 Servei d’Ecopatologia de Fauna Salvatge (SEFaS), Departament de Medicina i Cirurgia Animals, Universitat Autònoma de Barcelona (UAB), E-08193 Bellaterra, Barcelona, Spain; 3 Departament de Medicina i Cirugia Animals, Universitat Autònoma de Barcelona (UAB), E-08193, Bellaterra, Barcelona, Spain; 4 Espacio Natural Sierra Nevada, Carretera Antigua de Sierra Nevada, Km 7, E-18071 Pinos Genil, Granada, Spain; 5 Agencia de Medio Ambiente y Agua, Isla de la Cartuja, E-41092 Sevilla, Spain; 6 Estación Biológica de Doñana (CSIC), Av. Américo Vespucio, s.n, E-41092 Sevilla, Spain; 7 Área de Toxicología, Facultad de Veterinaria, Universidad de Murcia, 30100 Murcia, Spain

**Keywords:** Acetylcholinesterase, Bacteria, Lesion, Mangy skin, Non-dermal tissue, Sarcoptic mange, Serum amyloid a

## Abstract

**Background:**

Sarcoptic mange has been identified as the most significant infectious disease affecting the Iberian ibex (*Capra pyrenaica*). Despite several studies on the effects of mange on ibex, the pathological and clinical picture derived from sarcoptic mange infestation is still poorly understood. To further knowledge of sarcoptic mange pathology, samples from ibex were evaluated from histological, microbiological and serological perspectives.

**Methods:**

Samples of skin, non-dermal tissues and blood were collected from 54 ibex (25 experimentally infected, 15 naturally infected and 14 healthy). Skin biopsies were examined at different stages of the disease for quantitative cellular, structural and vascular changes. Sixteen different non-dermal tissues of each ibex were taken for histological study. Acetylcholinesterase and serum amyloid A protein levels were evaluated from blood samples from ibex with different lesional grade. Samples of mangy skin, suppurative lesions and internal organs were characterized microbiologically by culture. Bacterial colonies were identified by a desorption/ionization time-of-flight mass spectrometry system (MALDI TOF/TOF).

**Results:**

The histological study of the skin lesions revealed serious acanthosis, hyperkeratosis, rete ridges, spongiotic oedema, serocellular and eosinophilic crusts, exocytosis foci, apoptotic cells and sebaceous gland hyperplasia. The cellular response in the dermis was consistent with type I and type IV hypersensitivity responses. The most prominent histological findings in non-dermal tissues were lymphoid hyperplasia, leukocytosis, congestion and the presence of amyloid deposits. The increase in serum concentrations of acetylcholinesterase and amyloid A protein correlated positively with the establishment of the inflammatory response in mangy skin and the presence of systemic amyloidosis. A wide variety of bacterial agents were isolated and the simultaneous presence of these in mangy skin, lymph nodes and internal organs such as lungs, liver, spleen and kidney was compatible with a septicaemic pattern of infection.

**Conclusions:**

The alteration of biomarkers of inflammation and its implication in the pathogenesis of the disease and development of lesions in non-dermal tissues and septicaemic processes are serious conditioners for the survival of the mangy ibex. This severe clinical picture could be an important factor when considering the decision to eliminate animals that exceed a certain disease threshold from a population.

## Background

Sarcoptic mange is a highly contagious infection of the skin caused by the burrowing mite *Sarcoptes scabiei* that affects both humans and animals worldwide [1]. It is responsible for epizootic disease in several wild ungulate and carnivore species [2–4]. Severe outbreaks of sarcoptic mange have been reported in the Iberian ibex (*Capra pyrenaica*), some of which have led to high mortality rates [5, 6]. Although many studies have addressed sarcoptidosis in Iberian ibex in recent last years, the pathogenicity of the infection in this host is still not yet fully understood.

Clinical signs of sarcoptic mange generally include a combination of alopecia, scaling and crusting [7]. However, the severity and distribution of these lesions, as well as the outcome of the disease, vary between host species and individuals of the same species [1, 8]. Mild infestations often have little effect on hosts, although chronic infestations can affect fat reserves and food-conversion efficiency [9, 10]. Anemia, damage to inner organs and secondary bacterial complications may also compromise the survival of the host [11–13].

Despite the availability of wide-ranging information on the pathology of sarcoptic mange in the Iberian ibex [14–17], there are still few studies that address the pathological changes that occur in the skin and organs in mangy ibex. The objectives of this study were thus (i) to provide detailed histological descriptions of mangy skin at different stages of the disease; (ii) to assess histological changes in non-dermal organs in ibex with severe sarcoptic mange; (iii) to analyze the activity of markers of the inflammatory process and their implication in the pathogenesis of the disease; and (iv) to characterize microbiologically mangy skin and lesions as a means of identifying possible signs of secondary sepsis. To achieve these goals, we surveyed ibex that were both naturally and experimentally infected with *S. scabiei.*

## Methods

### Experimental facilities and animals

Thirty nine Iberian ibex (*C. p. hispanica*) (22 males and 17 females; 1–11 years of age) were captured, 33 in the Sierra Nevada Natural Space (SNNS) (36°55′ to 37°10′N, 2°56′ to 3°38′W), and 6 in the Sierras de Cazorla, Segura y Las Villas Natural Park (SCSVNP) (37°53′ to 37°88′N, 2°53′ to 2°88′W). Initial IgG levels against *S. scabiei* were measured by an enzyme-linked immunosorbent assay (ELISA) developed for alpine chamois (*Rupicapra rupicapra*) in order to exclude ibex that had previously been in contact with the disease [18]. Captured ibex were transferred to experimental facilities located in southern Spain (Las Mimbres, Sierra de Huétor Natural Park, 37°18′ to 37°30′N, 3°28′ to 3°47′W). They were kept in small groups (4–6 animals) in separate enclosures under observation during an eight-week acclimatization period. All ibex had *ad libitum* access to food and water [19, 20].

In addition, 15 naturally infested free-ranging ibex (nine males and six females; 4–9 years of age) were selectively harvested as part of a management program devoted to controlling both ibex density and the spread of sarcoptic mange in the SNNS.

All ibex were captured using a rifle and anaesthetic darts with a combination of ketamine (3 mg/kg) and xylazine (3 mg/kg) [21].

### Experimental infestation

After an adaptation period, 25 of the 39 ibex (22 from SNNS and three from SCSVNP) were experimentally infested with *S. scabiei*, and the remaining 14 ibex being left to act as controls. A 2cm^2^ skin fragment from a naturally *S. scabiei*-parasitized wild ibex from the SNNS was attached with elastic bandages to the previously shaved inter-scapular region to induce contact between the mites and the host skin. In order to determine the density and therefore the number of mites infesting each ibex, the density of mites was calculated in skin pieces adjacent to those used for the infestation. In these adjacent skin samples, mites were counted with a stereomicroscope after overnight digestion in 5% KOH solutions at 40 °C [22] and a thermal gradient induced by a light shone from below Petri dishes with black bottoms and transparent central areas [23].The resulting estimated dose received by each ibex was 750 ± 440 mites.

The experimental period lasted for 150 days after infestation. Experimentally and naturally infected ibex were visually assigned to one of the following three categories defined according to the percentage of skin surface affected: healthy (mange-free ibex), mild (initial and development stages, with lesions on < 50% of the host skin surface), and severe (consolidation and chronic stages with lesions on more than 50% of the host skin surface) [22].

### Blood and skin sample collection

Blood samples and skin biopsies were collected at 26, 46, 103 and 150 days post-infestation (dpi). Blood samples (20 ml) were taken from each ibex (both infested and control animals) by jugular venipuncture, and kept at 4 °C in a cooling box until reaching the laboratory. Adjacent skin biopsies were taken after shaving the inter-scapular region using an 8 mm diameter punch biopsy tool (KRUUSE® Biopsy Punch, Langeskov, Denmark). In the control group, skin samples were only collected at 103 dpi. To do so, each ibex was separated into a handling crush for subsequent physical restraint (blindfolding and limb immobilization) and a local anaesthetic was administered (ANESVET®, Ovejero Lab, León, Spain). After collecting the skin samples, each animal was treated with an antiseptic spray. Each biopsy was placed into 10% neutral buffered formalin for 48–72 h, and then transferred to 60% ethanol and stored at 4 °C until histological analysis.

### Necropsy and sample collection

Nineteen of the 25 ibex experimentally infected were euthanized at 150 dpi due to their severe mange infections. Naturally infected animals (all with more than 50% of their skin surface affected) were euthanized at the time of capture. Before euthanasia, blood samples were collected from each ibex. Animals were anaesthetized by intramuscular injection with a mixture of xylazine (3 mg/kg) and ketamine (3 mg/kg), and euthanized with T-61® (embutramide 12 mg/kg, membezonium iodide 3 mg/kg, tetracaine 0.3 mg/kg).

Scabietic ibex were necropsied under strict hygienic conditions immediately after death. Gross pathologic examination was performed on all animals and organs following a standard protocol. The following samples were collected from each ibex for histological study: superficial lymph nodes (SLN) draining the mangy skin lesions (submandibular, parotid, pre-scapular, sub-scapular, inguinal, mammary and popliteal), intracavitary lymph nodes (ILN) (mediastinal, mesenteric, hepatic, renal), central nervous system, tongue, thyroid gland, skeletal muscle, heart (papillary muscle), lungs (apical, middle and caudal lobes), liver, kidney, adrenal gland, spleen, pancreas, small intestine (ileum), large intestine (colon), ovary and testicle. All samples were fixed in a similar way to the skin biopsies. For the microbiological studies, the following samples were taken and stored separately in sterile tubes: skin (especially lesions of exudative dermatitis and/or abscesses), suppurative lesions, lungs, liver, spleen and kidney.

### Histopathology

Skin biopsies and the rest of the fixed non-dermal tissues were briefly washed with 10% phosphate buffered saline (PBS) solution and embedded in paraffin wax (EI LEICA TP1020 Automatic Tissue Processor®, Barcelona, Spain). Serial 5 μm sections from all specimens were mounted on glass slides (Super-Frost, Menzel-Gläser, Braunschweig, Germany), stained with haematoxylin and eosin (HE) and analyzed by light microscopy.

Skin sections were examined for quantitative cellular, structural and vascular changes (Table 1). Epidermal changes were evaluated in three randomly selected complete microscopic cross-sections. The final value was expressed as the average of the observations of the three sections. The counts of dermal inflammatory cell infiltrations (neutrophils, plasma cells, mast cells, eosinophils and lymphocytes) were performed in five randomly selected fields at 400× magnification in one section taken from the skin. The final number of cells was expressed as an average count at 400× HPF (high-powered fields). For the rest of the organs, three randomly selected cross-sections from each tissue were evaluated at 200× magnification.

**Table 1:** Median ± SD of histopathological changes assessed in sarcoptic mange skin lesions on the different days post--infection

	Healthy	26 dpi	46 dpi	103 s dpi	103 dpi	150 dpi
Epidermis						
Mites/burrows^1^	0.00	1.45 ± 0.82^a^	3.18 ± 1.25^b^	0.60 ± 1.57^a^	3.51 ± 6.18^b^	1.63 ± 1.68^a^
Basal cell hyperplasia/acanthosis^2^	5.00	15.54 ± 2.54^a^	12.77 ± 1.8^b^	9.95 ± 2.88^c^	13.80 ± 3.93^b^	12.22 ± 2.60^b^
Rete ridges^3^	0.00	17.08 ± 4.81^a^	13.10 ± 4.62^b^	7.40 ± 3.80^c^	14.40 ± 3.23^b^	15.01 ± 3.92^b^
Spongiotic oedema^4^	0.00	14.45 ± 3.85^a^	15.36 ± 3.29^a^	4.50 ± 3.77^c^	10.50 ± 3.83^b^	7.18 ± 5.54^b^
Serocellular crusts^5^	0.00	2.90 ± 0.54^a^	3.18 ± 0.60^a^	0.41 ± 0.69^b^	3.20 ± 1.13^a^	0.72 ± 0.64^b^
Eosinophilic crusts^6^	0.00	2.81 ± 0.60^a^	3.27 ± 0.78^a^	0.61 ± 0.70^b^	3.30 ± 0.67^a^	0.90 ± 0.83^b^
Exocytosis foci^7^	0.00	12.82 ± 4.19^a^	13.81 ± 3.91^a^	3.50 ± 3.06^c^	9.10 ± 3.51^b^	6.10 ± 3.76^b^
Apoptosis/necrosis^8^	0.00	12.27 ± 3.06^a^	14.54 ± 2.65^a^	2.90 ± 2.88^c^	9 ± 3.80^b^	6.08 ± 5.85^b^
Dermis						
Sebaceous glands	8.00	27.27 ± 3.70^a^	24.54 ± 3.32^a^	19.4 ± 6.10^b^	25.11 ± 6.65^a^	21.10 ± 3.17^b^
Lymphocytes	18.68	45.97 ± 11.85^a^	44.67 ± 12.14^a^	29.31 ± 6.48^b^	34.46 ± 13.40^b^	31.81 ± 90^b^
Eosinophils	0.20	8.49 ± 0.94^a^	11.40 ± 1.16^a^	2.02 ± 5.81^b^	4.23 ± 2.64^b^	5.14 ± 1.74^b^
Mast cells	0.10	6.72 ± 0.69^a^	8.32 ± 0.92^a^	1.14 ± 2.24^b^	3.42 ± 1.14^b^	2.53 ± 1.29^b^
Neutrophils	0.00	3.52 ± 4.48^a^	4.17 ± 1.76^a^	0.71 ± 1.27^c^	2.41 ± 3.39^b^	2.77 ± 3.62^b^
Plasma cells	0.00	0.53 ± 0.24^a^	0.64 ± 0.11^a^	0.37 ± 0.24^a^	0.42 ± 0.23^a^	0.43 ± 0.55^a^

^1^Number of mites and/or burrows

^2^Number of cells layers from the basal lamina to the stratum granulosum

^3^Number of rete ridges along the basal lamina

^4^Number of cells with hydropic degeneration

^5^Areas of transudation

^6^Areas of intense eosinophilic infiltration associated with thepresence of mites and/or burrows

^7^Number of exocytosis focus of inflammatory cells associated with vasodilation and vascular neoformation

^8^Number of cells with hyperchromatosis with karyorrhectic and pyknotic nuclei

*Notes*: On day 103 post-infection the ibex that showed self limiting clinic process were included separately (included in column 103 s dpi). Values that have different superscript letters (a-c) along the row differ significantly

### Acetylcholinesterase (AChE) and serum amyloid a (SAA) assay

Within 24 h of collection, serum was obtained by centrifugation at 4750× *g* for 10 min and stored separately at -82 °C. Serum SAA concentrations (μg/ml) were quantified as the mean value of two measurements using a commercially available ELISA kit (Tridelta Phase™ range serum amyloid, A Tridelta Development Ltd., Bray, Ireland). AChE activity (μmol/ml*min) was measured using acetylthiocholine as a substrate [24]. The method was adapted to an automatic analyzer [25].

### Microbiology

In the laboratory, samples were processed in biological safety cabinets within 12 h after collection. Twenty grams of each tissue and/or pathological material was placed in a sterile airtight bag with 3 ml of sterile 10% PBS and homogenized for 5 min (Stomacher 80 Biomaster®, Lardero, Spain). An aliquot of 800 μl of the resulting homogenate was added to a sterile cryotube together with 200 μl of glycerol. The cryo tubes were stored at -20 °C until microbiological analysis.

Samples were cultured on TSA and Columbia agar plates supplemented with 5% sheep blood (bioMérieux, Madrid, Spain) and incubated aerobically at 37 °C for 24–48 h. Cells from representative bacterial pure and freshly-cultivated colonies were re-suspended in 300 μl of HPLC-grade water and mixed vigorously prior to the addition of 900 μl of HPLC-grade ethanol. Subsequently, an acetonitrile/formic acid extraction protocol was performed following the manufacturer’s instructions (Bruker Daltonik, Bremen, Germany). After protein extraction, 1 μl of each isolate extract was spotted onto a 384-spot polished steel target plate, left to dry at room temperature and then overlaid with 1 μl of α-cyano-4-hydroxy-cinnamic acid (HCCA) matrix. All isolates were analyzed in a Bruker Daltonik UltrafleXtreme MALDI TOF/TOF (desorption/ionization time-of-flight mass spectrometry) system, which obtained one spectrum per sample used to assess the suitability of this spectrometric approach for identification at species level. Each spectrum was acquired using FlexControl software (Version 3.4) in automatic mode in a random sampling pattern. The identification of all the clinical isolates in this study was performed by MALDI Biotyper Real Time Classification software. The reliability of the identification was evaluated from the log (score) values, calculated with the MALDI Biotyper software mentioned above.

### Data analysis

The results of the histopathological changes and serum SAA and AChE concentrations were reported as median and standard deviations, calculated using routine descriptive statistical procedures. The Kolmogorov-Smirnov test was used to assess data normality. Non-parametric statistical methods were used to compare groups. A Mann-Whitney U-test was employed to compare the control group with the clinical stage of the exposed groups. To evaluate the response of the different parameters in term of the time of exposure and mange status of the different infested groups, Friedman tests with repeated measures were performed. In a second step, a Wilcoxon signed rank *post-hoc* comparison test with Bonferroni correction was used to determine the differences between the groups analyzed. A Spearman’s rank correlation coefficient test was applied to establish the coefficients between the different variables analyzed. To compare the frequency of lesions observed in non-dermal tissues between experimentally exposed and naturally infected animals, the Fisher’s exact test was used. *P*-values < 0.05 were considered to be statistically significant.

All statistical analyses were performed with the R software version 3. 3. 1 (R Development Core Team, December 2016) [26].

## Results

### Clinical signs

**Fig. 1:**
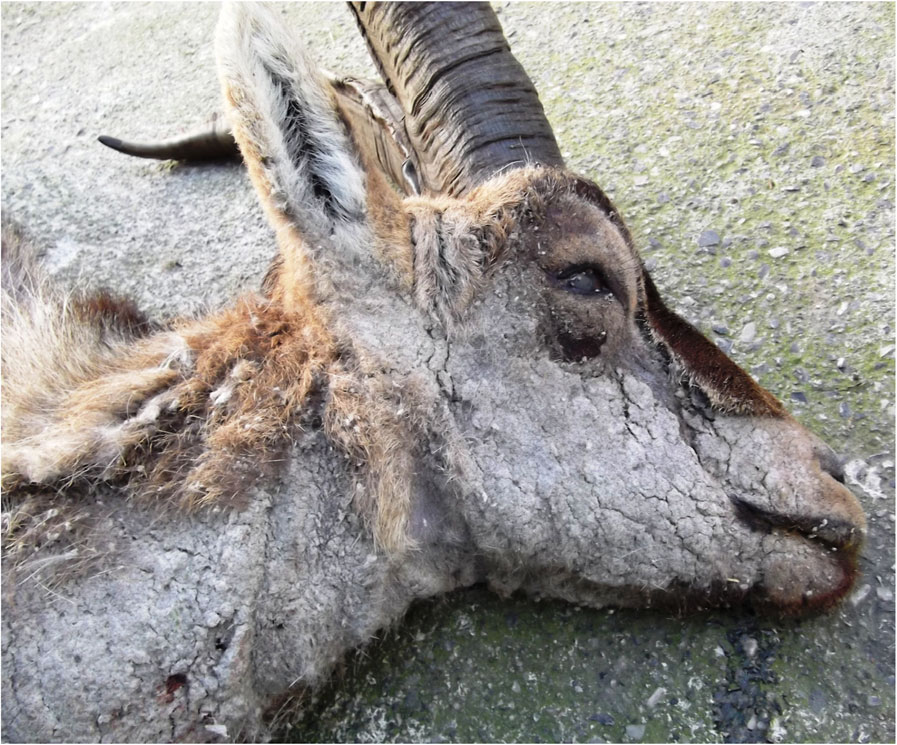
Iberian ibex with severe sarcoptic mange. Presence of thick crusts, scales and alopecia in facial area and neck

The clinical outcome and evolution of the infested ibex changed during the infestation period. Sixteen infested ibex progressed to severe stages of the disease. However, seven ibex, all of which from SNNS, only developed a self-limiting clinic process, with mange lesions on less than 50% of their skin surface, and showed evident signs of recovery. All infested ibex from SCSVNP evolved to severe stages of the disease. Until 46 dpi, all ibex developed localized lesions in the inoculation zone (inter-scapular area) and facial area (mildly affected). From 103 dpi onwards, in all ibex except those with a self-limiting clinic process, mange lesions were generalized, extending over the lumbar region and the ventral part of the abdomen and limbs (severely affected). Animals showed pruritus throughout the disease and intermittently scratched themselves. In the most severe clinical stages, both naturally and experimentally infested ibex showed scales, marked lichenification, alopecia, crusts, dermal fissures in limbs and moderate inflammation of eyelids and lips (Fig. 1). In the terminal phase, animals showed weakness, prostration and weight loss.

### Histopathological and microbiological findings

**Fig. 2:**
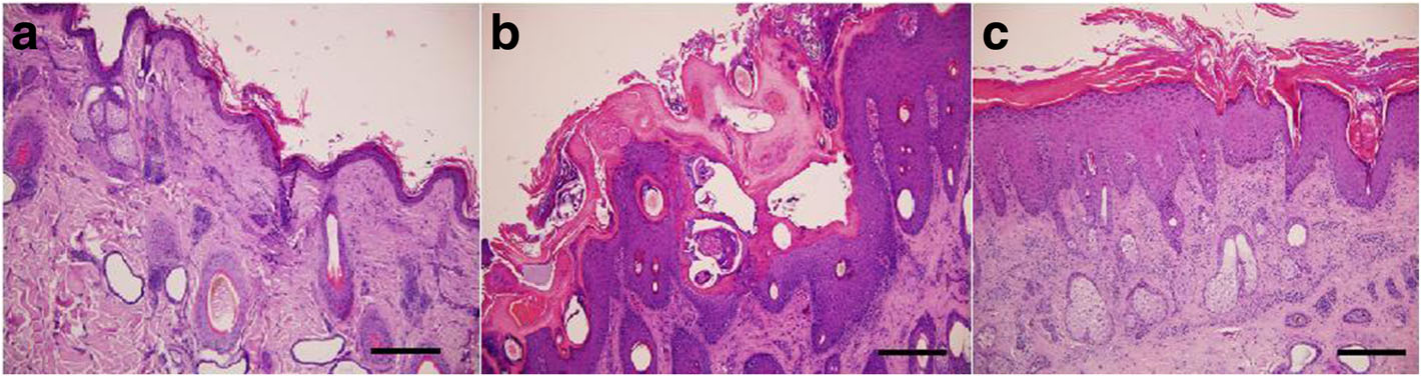
Light micrographs of ibex skins. **a** Section of uninfected ibex skin. **b** Skin section at 46 dpi. Presence of mites, parakeratotic hyperkeratosis, acanthosis, serocellular and eosinophilic crusts and keratinocytes with spongiotic oedema. **c** Skin section at 150 dpi. Uniform corneal layer with lesser orthokeratotic hyperkeratosis, severe acanthosis and rete ridges and predominance of mononuclear inflammatory infiltrate *Scale-bars*:251 μm

Histopathological changes in mangy skin between dpi were compared and shown in Table 1. Such changes were detected in all of the analyzed stages, both in the ibex that progressed to more severe stages and in those with a self-limiting clinic process, and always in amounts significantly (*P* < 0.0001) higher than those of the control group (Fig. 2a). For the epidermis, the median values of mites/burrows varied between different dpi (Friedman’s test: *χ* ^2^ = 93.94, *df* = 2, *P* = 0.022), increasing significantly (*post-hoc* test: *P* = 0.012) between 46 and 103 dpi. In the final stage the values decreased and returned to the levels of the early stages of the disease. The number of eosinophilic and serocellular crust foci were correlated (*r*_(353)_ = 0.546, *P* < 0.0001 and *r*_(384)_ = 0.402, *P* = 0.0012, respectively) with the presence of mites/burrows, but values were significantly (*post-hoc* tests: *P* = 0.012 and *P* = 0.0093, respectively) lower by 150 dpi. Acanthosis (Friedman *F*_*r*_ test: *χ* ^2^ = 98.04, *df* = 2, *P* = 0.003) and rete ridges (Friedman *F*_*r*_ test: *χ* ^2^ = 98.84, *df* = 2, *P* = 0.002) showed variation along the different dpi. These reached their maximum number (*post-hoc* tests: *P* = 0.032 and *P* = 0.044, respectively) by 26 dpi and then decreased significantly (*post-hoc* tests: *P* = 0.012 and *P* = 0.022, respectively) by 46 dpi, remaining unchanged during the rest of the disease. Likewise, acanthosis was correlated (*r*_(394)_ = 0.329, *P* = 0.031) with the presence of mites/burrows. Signs of cytotoxicity such as spongitic oedema, apoptotic keratinocytes and exocytosis foci increased significantly (*post-hoc* test: *P* < 0.0001) up to 46 dpi (Fig. 2b), and then decreased significantly (*post-hoc* test: *P* < 0.0001) during the more severe stages (from 103 dpi onward) (Fig. 2c). In ibex with self-limiting lesions, all epidermal changes were significantly (*post-hoc* test: *P* < 0.0001) lower than those that progressed to severe stages (103and 150 dpi), and were even lower than those observed during the first days of the disease (26 dpi).

In the dermis, the number of sebaceous glands also varied according to dpi (Friedman *F*_*r*_ test: *χ* ^2^ = 90.14, *df* = 2, *P* = 0.036), remaining unchanged until 150 dpi, at which point their number fell significantly (*post-hoc* test: *P* = 0.044). The number of lymphocytes (*r*_(343)_ = 0.586, *r*_(346)_ = 0.584, *r*_(286)_ = 0.640), eosinophils (*r*_(431)_ = 0.284, *r*_(398)_ = 0.324, *r*_(452)_ = 0.205), mast cells (*r*_(277)_ = 0.763, *r*_(281)_ = 0.699, *r*_(149)_ = 0.811) and neutrophils (*r*_(293)_ = 0.699, *r*_(354)_ = 0.566, *r*_(366)_ = 0.515) was significantly correlated (*P* < 0.0001) with the presence of spongiotic oedema, exocytosis foci and apoptotic cells respectively. With the exception of plasma cells (Friedman *F*_*r*_ test: *χ* ^2^ = 10.96, *df* = 2, *P* = 0.067), the number of lymphocytes, mast cells, eosinophils and neutrophils was significantly (*P* = 0.0023, *P* = 0.012, *P* = 0.0087, *P* = 0.0023, respectively) higher during the first 46 dpi, but then decreased significantly (*P* < 0.001). The number of plasma cells was low in all the stages analyzed. In animals with self-limiting lesions, the number of inflammatory cells decreased significantly (*post-hoc* test: *P* < 0.0001) in relation to the first 46 dpi. However, except for neutrophils (*post-hoc* test: *P* = 0.019), these values showed no significant differences (*post-hoc* test: *P* = 0.394) with the ibex that reached the most severe stages of the disease.

Other changes observed (not shown in Table 1) include: mixed hyperkeratosis (ortho-and parakeratotic) with a predominance of the orthokeratotic form 46 dpi, bacterial colonies on the surface, mite feces, dermal fibrosis from the basal lamina to secretory duct of the sebaceous glands, multifocal deposits of melanin at the level of the basal lamina, pilose follicles in the catagen phase, and the dilation of the apocrine glands. Folliculitis was only detected in one ibex.

The histopathological findings in the non-dermal tissues are detailed in Table 2. Samples from experimentally and naturally infested ibex were evaluated together, due to the similar clinical picture of these animals. No differences (Fisher’s exact test: *P* > 0.087; OR: 0.579; 95% CI: 0.342–0.975) were found between the lesions observed in the two groups.

**Table 2:** Description of necropsy findings in the non-dermal tissue of the Iberian ibex affected by severe sarcoptic mange

Organs	*n*	Gross pathologic examination	Microscopical findings
Superficial LN	206	Lymphadenomegaly (two and three times the normal size).	Lymphoid hyperplasia with activation of LF, formation of germinal centers and increase in lympho-plasma cells and macrophages in cords and medullary sinuses (Serous lymphadenitis) (+++)
		Purulent lesion (7.76%) (Fig. 3)	Purulent lymphadenitis (7.76%) (+++)
		Edema	Congestion and edema (+++)
		Congestion	Subcapsular, follicular and medullary amyloidosis (71.35%) (++)
Deep LN	105	Lymphadenomegaly (less than twice the normal size)	Histological pattern similar to superficial LN (++)
		Edema	Subcapsular, follicular and medullary amyloidosis (75.23%) (++)
		Congestion	*Sarcocystis* spp. (mesenteric LN) (12.38%) (+)
CNS	12	Subdural congestion	Gliosis and perivascular cuffs of mononuclear cell (25.00%) (+)
Tongue	34	None	*Sarcocystis* spp. with cellular infiltration (lymphocytes, mast cells and eosinophils) (58.82%) (+)
Thyroid gland	34	None	None
Skeletal muscle	34	None	*Sarcocystis* spp. with monocuclear infiltration (32.35%) (+)
			Mononuclear myositis in the absence of parasites (14.74%) (+)
Heart	34	Hydropericardium (82.35%)	*Sarcocystis* spp. (44.11%) (+)
		Absence of pericardial fat	Mononuclear myocarditis (monocytes, lymphocytes and plasma cells) with infiltration of adipose cells (23.52%) (+)
		Chicken fat clot (35.29%)	Muscle mineralization (5.88%) (+)
Lungs	102	Interstitial emphysema in apical lobes (41.17%)	Granulomatous inflammation with presence of intra- alveolar parasites and infiltration of eosinophils, neutrophils and lymphocytes (verminous pneumonia) (88.23%) (+++)
		Fibrotic nodular lesions in caudal lobes (88.23%)	Infiltration of neutrophils, eosinophils, macrophages and lymphocytes, congestion, edema and areas of necrosis (bacterial pneumonia) (8.82%) (+++)
			Infiltration of neutrophils, eosinophils, macrophages and lymphocytes, congestion, edema and areas of necrosis (bacterial pneumonia) (8.82%) (+++)
Abdominal cavity	34	Ascitic fluid (transudate)	
		Hepatoperitoneal cysticercosis	
Liver	34	Congestion	Perivascular amyloid deposits in portal triad and hepatic sinusoids (11.76%) (++)
		Increased size	Congestion (+++) and leukocytosis (67.64%) (++)
		Fibrosis (8.82%)	Parasitic fibrosis (8.82%) (+)
Kidney	68	Absence of perirenal fat	Amyloid deposits in glomerular mesangium (amyloid nephrosis) and cortical and medullary tubular interstitium with decreased capillary lumen (chronic interstitial nephritis and ischemic tubular atrophy) (20.58%) (++)
		Congestion	Mesangial thickening, tubular mineralization and leukocytosis (50.00%) (+)
Adrenal gland	68	Increased size	Amyloid deposits in cortex and adrenal medulla (26.47%) (++)
		Increased color	Leukocytosis (5.88%) (+) and adrenal cortical hypoplasia (17.64%) (+++)
Spleen	34	Increased size	Hyperplasia of LF with formation of germinal centers (+++)
		Lymphoid hyperplasia	Amyloid deposits in LF and PLS of the white pulp and the splenic cords and venous sinuses of the red pulp “sago spleen” (70.58%) (+++)
		Congestion	Congestion (+++) and leukocytosis (+)
Pancreas	26	None	Amyloid deposits in exocrine pancreas (15.38%) (+) and leukocytosis (+)
Intestine	66	Congestion	Chronic parasitic enteritis (21.21%) (++) Amyloid deposits in MALT (25.75%) (+)
Testicle	7	None	None
Ovary	32	None	None

The table includes the number of samples analyzed (*n*) and the detection rate (%). The generalized findings do not show the detection rate. Each change was scored from + to +++, where + = focal, ++ = multifocal, +++ = generalized

*Abbreviations*: LN, lymph node; LF, lymphoid follicle; CNS, central nervous system; BALT, bronchus-associated lymphoid tissue; PLS, peri-arteriolar lymphoid sheaths; MALT, mucosa-associated lymphoid tissue

### Biomarkers of inflammation

With respect to the healthy group, AChE concentrations increased significantly (Wilcoxon rank sum test: *W* = 78, *P* = 0.0037) in the ibex with localized lesions (with maximum values at 46 dpi) and, by contrast, decreased in severely infected animals (Wilcoxon rank sum test: *W* = 93, *P* = 0.015) (Table 3). AChE values were positively correlated with the number of lymphocytes (*r*_(374)_ = 0.531, *P* = 0.032), eosinophils (*r*_(388)_ = 0.343, *P* = 0.042), mast cells (*r*_(363)_ = 0.398, *P* = 0.0076) and neutrophils (*r*_(452)_ = 0.252, *P* = 0.011) found in mangy skin lesions. SAA concentrations only increased significantly (Wilcoxon rank sum test: *W* = 6.5, *P* < 0.0001) in the most severe stages of the disease (Table 3). SAA values were negatively correlated (*r*_(496)_ = −0.184, *P* = 0.0087) with AChE concentrations. At the time of euthanasia, serum SAA levels were positively correlated (*r*_(282)_ = 0.719, *P* < 0.0001) with the number of organs that showed reactive amyloidosis (Table 4).

**Table 3:** Median ± SD (range) of serum SAA and AChE concentrations in Iberian ibex according their sarcoptic mange status

	Healthy	Mildlly affected	Severely affected
SAA (µg/ml)	4.45 ± 6.47^a^ (1.50–26.80)	4.72 ± 11.52^a^ (1.50–51.40)	11.61 ± 13.47^b^ (1.51–80.40)
AChE (μmol/ml*min)	0.29 ± 0.12^a^ (0.21–0.80)	0.42 ± 0.19^b^ (0.20–0.12)	0.33 ± 0.17^c^ (0.10–0.90)

*Note*: Values that have different superscript letters (a-c) along the row differ significantly

**Table 4:** Median ± SD (range) of serum SAA concentrations in relation to the percentage of amyloidosis detected in the total number of organs analyzed per ibex (*n* = 25)

	Not detected	< 25%	25–50%	> 50%
SAA (**μ**g/ml)	5.05 ± 4.67^a^ (1.50–9.50)	9.50 ± 4.61^b^ (1.51–10.80)	14.35 ± 7.44^c^ (9.30–31.30)	21.65 ± 9.97^d^ (11.40–51.40)

*Note*: Values that have different superscript letters (a-d) along the row differ significantly

### Microbiology

The different microbiological agents isolated on mangy skin, lesions and other tissues are shown in Table 5. The evaluation of the accuracy of MALDI-TOF MS was determined by calculating its sensitivity and specificity for each of the isolated species. The sensitivity and specificity values were 100% (95% CI: 40.7–100 and 89.8–100, respectively, depending on the species). All identifications of the isolated agents included in Table 5 are considered to be consistent and reliable, with an average score and range of 2.230 (2.134–2.875) (MALDI Biotyper Real Time Classification software).

**Table 5:** Microbiological agents isolated by the MALDI-TOF MS method in Iberian ibex (*n* = 34) affected by severe sarcoptic mange

Mangy skin (*n* = 34)		Superficial suppurative lesions (*n* = 16)^a^		Other tissues (*n* = 43)	
Agent	%	Agent	%	Agent	%
*Bacillus altitudinis*	5.88	*Aerococcus viridians*	6.25	Lungs (*n* = 12)	
*Corynebacterium glutamicum* ^c^	2.94	*Corynebacterium glutamicum* ^c^	6.25	*Pasteurella multocida*	25.00
*Escherichia coli*	14.70	*Staphylococcus aureus*	43.75	*Staphylococcus aureus*	25.00
*Proteus vulgaris*	11.76	*Staphylococcus warneri*	25.00	*Staphylococcus sciuri*	8.33
*Pseudomonas aeruginosa*	17.64	*Staphylococcus simulans*	7.69	*Staphylococcus warneri*	25.00
*Staphylococcus aureus*	52.92	*Staphylococcus xylosus*	7.69	*Streptococcus lutitiensis*	16.66
*Staphylococcus warneri*	29.41	*Staphylococcus nepalensis* ^b^	6.25	Liver (*n* = 11)	
*Staphylococcus sciuri*	8.82	*Streptococcus ovis*	18.75	*Staphylococcus aureus*	36.36
*Staphylococcus simulans*	5.88	*Trueperella pyogenes*	25.00	*Staphylococcus xylosus*	18.18
*Staphylococcus chromogenes*	2.94			*Streptococcus lutitiensis*	27.27
*Staphylococcus xylosus*	5.88			Spleen (*n* = 10)	
*Staphylococcus nepalensis* ^b^	5.88			*Escherichia coli*	20.00
*Streptococcus pluranimalium*	2.94			*Staphylococcus aureus*	30.00
*Streptococcus lutitiensis*	5.88			*Streptococcus warneri*	20.00
				Kidney (*n* = 10)	
				*Escherichia coli*	20.00
				*Staphylococcus aureus*	40.00
				*Staphylococcus xylosus*	20.00
				*Streptococcus lutitiensis*	20.00

The table includes the number of samples analyzed (*n*) and detection rate (%)

^a^Suppurative lesions detected in superficial lymph nodes and subcutaneous tissue

^b^Isolated only in naturally infected ibex

^c^Isolated only in experimentally infected ibex

## Discussion

The present study reveals structural changes in the skin and several internal organs in Iberian ibex affected by sarcoptic mange, and shows a relationship between pathological findings and the systemic inflammatory response. Additionally, mangy skin and lesions associated with *S. scabiei* infection were characterized microbiologically.

The macroscopic and histological skin lesions observed in mangy ibex closely match the classical descriptions of sarcoptic mange in wild and domestic animals [1, 3]. The cellular response was dominated largely by mononuclear cells, eosinophils and mast cells (intact and degranulated) up to 46 dpi. Subsequently, the number of cells fell significantly from 103 dpi onward. Our observations agree with those in red foxes (*Vulpes vulpes*) [8], raccoon dogs (*Nyctereutes procyonoides*) [27], wombats (*Vombatus ursinus*) [28] and Eurasian lynx (*Lynx lynx*) [29]. However, the infiltration of eosinophils and mast cells was not as intense as in the above mentioned species and was similar to the findings previously described in wild boars [30], which may explain the presence of less obvious marked hyperkeratosis. According to our results, ibex showed signs that are compatible with a combined type I (immediate) and type IV (delayed) hypersensitivity reaction to infection [31]. We thus propose that up to 46 dpi, the histological pattern corresponded to type I hypersensitivity since cytotoxicity signs were observed (Fig. 2b) and correlated positively with the number of cells. Subsequently, these signs decreased along withthe cellular infiltrate which suggests type IV hypersensitivity (Fig. 2c).This conclusion needs to be demonstrated by future studies by the immune-histochemical isotype of the lymphocytes or by the expressed cytokine pattern. The neutrophilic response could be due not only to the presence of the mites, but also to secondary bacterial infections or excoriations. As in all other species, the number of plasma cells was low, which may be linked to a reduced role of the humoral response in the infection. We can therefore say that the overexpression of eosinophils, mast cells and neutrophils was associated with a detrimental and non-protective response that was unable to control the infection. This was shown by the observations of ibex with self-limiting clinic process. The presence of abundant serocellular crusts is apparently a mechanism that destroys mites or inhibits their burrowing into the skin as these crusts contain specific antibodies and other toxic components that can intoxicate mites [32]. The hypertrophy and dilation of sebaceous and apocrine glands could be due to the obstruction of the excretory duct of glands by the hyperkeratotic crusts. Finally, the orto- and parakeratotic scales in the stratum corneum correspond to the previous passage of mites through the incompletely differentiated layers of the epidermis.

Cholinesterases have been reported as having the capacity to increase local and systemic inflammatory events in various pathological states [33]. In our study we observed an increase in AChE levels in the localized form of the disease in experimentally-infested ibex (Table 3), which coincides and correlates significantly with the increase in inflammatory activity observed in the mangy skin during the first 46 dpi. Our results are similar to those described in canine demodicosis [34] and reinforce the idea that *S. scabiei* infection alters the cholinergic system and thereby contributing to the establishment of the local inflammatory response and the pathogenesis of the disease.

**Fig. 3:**
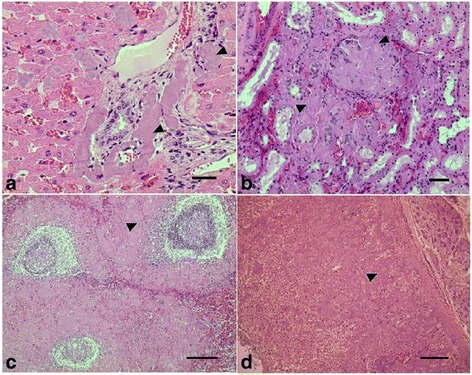
Light micrographs of ibex non-dermal organs. **a** Liver. Perivascular amyloid deposits in portal spaces and hepatic sinusoids (arrowheads) and congestion. **b** Kidney. Loss of glomerular structure due to the presence of amyloid deposits (arrowheads) at the mesangial level with a reduction of urinary spaces (amyloid nephrosis) and at the level of cortical tubular interstitium with decreased capillary lumens (chronic interstitial nephritis and ischemic tubular atrophy). **c** Spleen. Amyloid deposits (arrowhead) at the level of the mantle and crown of the Malpigian follicles and peri-arteriolar lymphoid sheaths of the white pulp, extending to splenic cords and venous sinuses of the red pulp (“Sago spleen”).**d** Adrenal gland. Loss of adrenal structure due to the presence of amyloid deposits (arrowhead) at the glomerular and fascicular levels of the adrenal cortex. *Scale-bars*: **a**, 62.7 μm; **b**, 62.7 μm; **c**, 251 μm; **d**, 251 μm

The most prominent histological findings in non-dermal tissues were lymphoid hyperplasia, leukocytosis, congestion, and the presence of amyloid deposits (Table 2). Systemic amyloidosis often occurs secondarily to chronic inflammatory or necrosis as is the case of sarcoptic mange [11, 35]. Serum SAA is an acute phase protein of inflammation [36]. In our study, serum SAA concentrations increased significantly in the most severe phase of the disease (Table 3), which coincides with the description by Ráez-Bravo [37]. In addition, higher levels were present in ibex in which more tissues with amyloidosis were detected (Table 4). This is because increased levels of this protein lead to a precipitation of its insoluble forms and turn it into a pathological protein [38]. A secondary effect of amyloid deposits could be the atrophy and necrosis of juxtaposed cells due to pressure (Fig. 3). Such lesions could be involved with changes in the serology and blood chemistry reported in mangy ibex [17]. Additionally, leukocytosis could be attributable to the cellular immune response, tissue response to damage or necrosis caused by inflammation or septicaemia. Generalized lymphadenomegaly, together with lymphoid hyperplasia, are congruent with a cell-mediated immune response [3], although it could also be related to secondary infections of the damaged skin. Among the gross findings, transudate fluid was found in the pericardium and the abdominal cavity. This may have been caused by hypoproteinemia associated with disease [17], or by liver or kidney lesions derived from amyloidosis. Finally, pericardial effusion could trigger restrictive cardiac insufficiency that could explain the generalized congestion that was noted, as well as the weakness and prostration described in some ibex.

A wide variety of bacterial agents were isolated in mangy ibex (Table 5). The simultaneous presence of these pathogens in mangy skin, and also suppurative lesions in lymph nodes and in various organs is compatible with a septicaemic pattern of infection [12, 39]. The bacteria identified were similar to those previously described in psoroptic mange and other skin diseases in livestock [40, 41]. *Staphylococcus* was the dominant genus and infrequent species such as *S. nepalensis* were isolated. Infections by these bacteria are often secondary to a primary skin disease that provides the conditions for these commensal bacteria to proliferate [40, 42, 43]. Under these conditions, some bacterial strains can produce diverse dermotoxins that can aggravate the skin injuries produced by mites [44, 45]. Further toxicity studies are necessary if we are to specify the type of toxins expressed in mangy ibex. It has also been demonstrated that the colonization of skin lesions due to *Psoroptes ovis* with *S. aureus* causes a specific response of IgG antibodies against *S. aureus* antigens, suggesting that these bacteria may play some role in the immune-pathogenesis of mange [46]. The mites themselves could contribute to the spread of these pathogenic bacteria since various strains of *Staphylococcus* have been isolated in mite burrows and in their faecal pellets [47]. However, it has been reported that both mites and the skin bacteria produce molecules that inhibit the pathways of the complement system and stimulate the production of various cytokines and, thereby, promoting bacterial survival and growth as well as the chemotaxis of inflammatory cells [13, 48].

In humans, especially in children, scabies-associated skin bacterial infection is a common risk factor for systemic complications such as acute post-streptococcal glomerulonephritis and sometimes rheumatic heart disease [49, 50]. However, in mangy ibex, no necrotic foci, abscesses or signs of inflammation associated with bacteremia were found in the kidneys, livers, spleens or lungs. This thereby indicates that the lympho-hematogenus dissemination of these pathogens a priori has fewer serious consequences than in other species [11, 12, 29, 39]. The areas of myocarditis and gliosis detected have also been described in other species with sarcoptic mange [29, 39], although in our case we cannot assume that this was due to the septicaemic state. From a veterinary perspective, the administration of antibiotic substances should be considered, in addition to antiparasitic treatment, in the individual treatment of sarcoptic mange in the Iberian ibex.

## Conclusion

In this study we provide a complete description of sarcoptic mange in the Iberian ibex through a description of its effects in naturally and experimentally infested animals, combining a serological, microbiological and histological approach. The alteration of biomarkers of inflammation and its implication in the pathogenesis of the disease and development of lesions in non-dermal tissues and septicaemic processes are serious conditioners for the survival of mangy ibex. These findings could be relevant in the management of affected ibex populations, since this could contribute to decisions on the elimination of animals that exceed a certain disease threshold from a population. In turn, our work provides a solid basis for further investigation of other factors that influence the pathogenesis of this disease.
